# Limited T Cell Receptor Repertoire Diversity in Tuberculosis Patients Correlates with Clinical Severity

**DOI:** 10.1371/journal.pone.0048117

**Published:** 2012-10-26

**Authors:** Wei Luo, Jin Su, Xiao-Bing Zhang, Zhi Yang, Ming-Qian Zhou, Zhen-Min Jiang, Pei-Pei Hao, Su-Dong Liu, Qian Wen, Qi Jin, Li Ma

**Affiliations:** 1 Institute of Molecular Immunology, School of Biotechnology, Southern Medical University, Guangzhou, People’s Republic of China; 2 Department of Respiratory Diseases, Nanfang Hospital, Southern Medical University, Guangzhou, People’s Republic of China; 3 Institute of Pathogen Biology, Chinese Academy of Medical Sciences & Peking Union Medical College, Beijing, People’s Republic of China; University of Palermo, Italy

## Abstract

**Background:**

The importance of CD4^+^ and CD8^+^ T cells in protection against tuberculosis (TB) is well known, however, the association between changes to the T cell repertoire and disease presentation has never been analyzed. Characterization of T-cells in TB patients in previous study only analyzed the TCR β chain and omitted analysis of the Vα family even though α chain also contribute to antigen recognition. Furthermore, limited information is available regarding the heterogeneity compartment and overall function of the T cells in TB patients as well as the common TCR structural features of Mtb antigen specific T cells among the vast numbers of TB patients.

**Methodology/Principal Findings:**

CDR3 spectratypes of CD4^+^ and CD8^+^ T cells were analyzed from 86 patients with TB exhibiting differing degrees of disease severity, and CDR3 spectratype complexity scoring system was used to characterize TCR repertoire diversity. TB patients with history of other chronic disease and other bacterial or viral infections were excluded for the study to decrease the likely contribution of TCRs specific to non-TB antigens as far as possible. Each patient was age-matched with a healthy donor group to control for age variability. Results showed that healthy controls had a normally diversified TCR repertoire while TB patients represented with restricted TCR repertoire. Patients with mild disease had the highest diversity of TCR repertoire while severely infected patients had the lowest, which suggest TCR repertoire diversity inversely correlates with disease severity. In addition, TB patients showed preferred usage of certain TCR types and have a bias in the usage of variable (V) and joining (J) gene segments and N nucleotide insertions.

**Conclusions/Significance:**

Results from this study promote a better knowledge about the public characteristics of T cells among TB patients and provides new insight into the TCR repertoire associated with clinic presentation in TB patients.

## Introduction

Tuberculosis (TB) is a common, worldwide contagious infection caused by infections with Mycobacterium tuberculosis (Mtb) [1]. Mtb is an intracellular bacterium that can be cleared following the elicitation of effective cell-mediated immune responses [2], [3]. Control of Mtb infections is mediated primarily by the development of T helper 1 (Th1) type immune response that involves the participation of CD4^+^, CD8^+^ T lymphocytes, and macrophages [4]–[6]. The central role of CD4^+^ T cells in protection against TB has been clearly demonstrated by the increased susceptibility to Mtb infections in HIV/AIDS patients in correlation with diminished CD4^+^ T cell counts [7], [8]. However, strong CD4^+^ T cell responses cannot alone resolve TB, that is, CD8^+^ T cells are also necessary based on *in vivo* experiments carried out in murine Mtb infection models [9]–[11]. Mtb-reactive CD8^+^ T cells have also been reported to be present at high frequencies in the circulation of patients with active tuberculosis, further supporting a role for CD8^+^ T cells in mediating immunity to Mtb [12].

**Table 1:** Baseline characteristic and CDR3 scores of TB patients.

Characteristic:	Mild group (n = 25)	Moderate group (n = 28)	Severe group (n = 33)	*P* values
**Age in years**				*0.807*
Median	44.0±14.04	44.96±14.72	42.58±14.63	
Range	21–69	19–73	18–74	
**Sex - no. (%)**				*0.834*
Male	16 (64)	20 (71.4)	24 (72.7)	
Female	9 (36)	8 (28.6)	9 (27.3)	
**BCG vaccine- no. (%)**				*0.816*
Yes	11	10	15	
No	10	13	12	
Indeterminacy	4	5	6	
**Tuberculin skin test- no. (%)**				*0.451*
0–5 mm	5	6	5	
5–10 mm	8	8	7	
10–15 mm	7	10	13	
>15 mm	5	4	8	
**previous TB or TB contact - no. (%)**				*0.400*
Yes	7	10	16	
No	5	6	4	
Unknown	13	12	13	
**smoking status- no.**				*0.432*
Non smoker	13	16	14	
Ex-trivial smoker (<1/day)	3	4	2	
Trivial smoker <1 cig/day	6	3	11	
Light smoker - 1–9 cigs/day	3	5	6	
**Type of TB- no. (%)**				*0.174*
Pulmonary tuberculosis	12 (48)	15 (53.57)	13 (39.40)	
Tuberculous pleurisy	12 (48)	11 (39.29)	11 (33.33)	
Chronic fibrocavitative pulmonary tuberculosis	1(4)	1(3.57)	4(12.12)	
Infiltrates tuberculosis	0	1(3.57)	4(12.12)	
hematogenous disseminated pulmonary tuberculosis	0	0	1 (3.03)	
**CDR3 spectratype complexity score**				
CD4^+^ T-cell				
Vα	249.32±8.20	231.5±9.33	216.18±15.25	*0.000*
Vβ	169.76±6.67	168.11±5.63	165.58±5.81	*0.032*
CD8^+^ T-cell				
Vα	221.44±16.97	207.86±21.45	194.45±22.27	*0.000*
Vβ	152.48±14.69	151.82±10.52	147.67±11.97	*0.268*
**Relative complexity score**				
CD4^+^ T-cell				
Vα	0.95±0.03	0.88±0.03	0.82±0.06	*0.000*
Vβ	0.99±0.04	0.98±0.03	0.96±0.03	*0.001*
CD8^+^ T-cell				
Vα	0.89±0.07	0.83±0.08	0.78±0.09	*0.000*
Vβ	0.91±0.09	0.91±0.06	0.89±0.07	*0.136*

To date, the importance of CD4^+^ and CD8^+^ T cells in protection against TB appears clear [13], [14], clonally expanded CD8^+^ T cells has been found in granuloma lesions and PBMCs in Mtb-infected individuals [15], [16]. However, the heterogeneity compartment of the CD4^+^ and CD8^+^ T cells in TB patients as well as the influence of TB disease severity on the CD4^+^ and CD8^+^ T cell receptor (TCR) repertoire diversity has never been analyzed.

The Mtb antigen specific TCR gene modified CD4^+^ and CD8^+^ T cells has been developed for autologous adoptive T cell immunotherapy study [17]. In an attempt to identify and characterize Mtb antigen specific T cells among the vast numbers of TB patients [18], it is important to detect Mtb antigen specific T cells which share TCR structural features, i.e., biased utilization of particular TCR variable gene fragment or contain a public complement determining region 3 (CDR3) motif [19], [20]. But the common TCR structural features of Mtb antigen specific T cells among the vast numbers of TB patients are rarely reported.

In this study, we analyzed the CD4^+^ and CD8^+^ TCR repertoire from 86 TB patients with differing levels of disease severity. Many factors influence the TCR repertoire of each individual including antigen stimulation, age, and chemotherapy etc. The extent of the likely contribution of TCRs specific to non-TB antigens is not known. In order to reduce the impaction of non-TB antigens to the TCR repertoire as far as possible, TB patients had chronic disease history or with other bacterial or viral (include HIV) infections, and patients accepted chemotherapy or other biological treatment in the past five years were excluded for the study.

**Figure 1:**
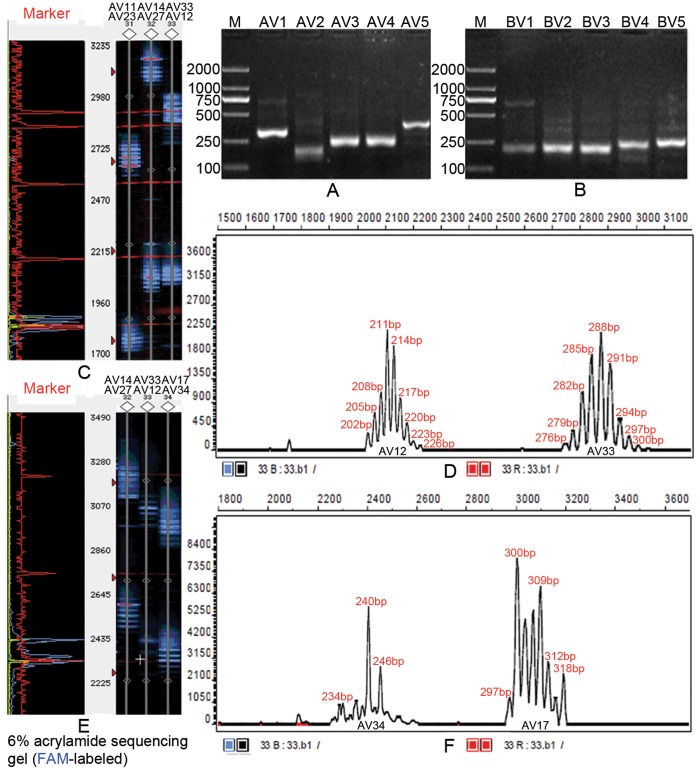
GeneScan results of partial TCR Vα and Vβ CDR3 products of CD4^+^ T cell. (A) PCR products of TCR Vα1-Vα5 of TB patient-1 (mild group) analyzed on a 1.5% agarose gel stained with ethidium bromide. (B) PCR products of TCR Vβ1–Vβ5 of TB patient-1 analyzed on 1.5% agarose gels. (C) Partially fluorescent PCR products of TCR Vα of healthy control-1 analyzed on a 6% acrylamide sequencing gel. (D) CDR3 size and fluorescence intensity analysis of Vα2 and Vα33 of healthy control-1. (E) Partially fluorescent PCR products of TCR Vα of TB patient-1 analyzed on a 6% acrylamide sequencing gel. (F) CDR3 size and fluorescent intensity analysis of Vα17 and Vα34 of TB patient-1.

**Figure 2:**
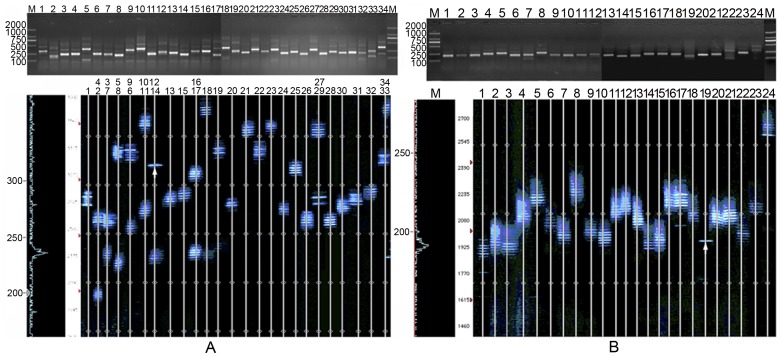
CDR3 spectratypes of TCR families for CD8^+^ T cells of TB patient-63 (severe group). (A**)** The numbers (1–34) denote the corresponding PCR products for TCR families Vα1–Vα34. In the sequencing gel, Lanes 2–7, 10, 20, and 25 include two TCR Vα families in one line. (B) The numbers (1–24) denote the corresponding PCR products of TCR families Vβ1–Vβ24. The lanes with only one bands marked with white arrow.

**Figure 3:**
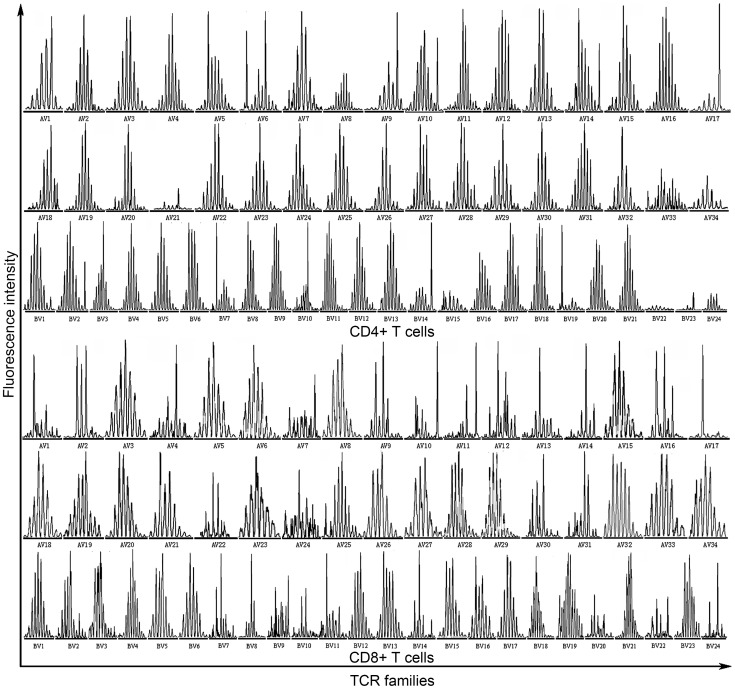
CDR3 spectratypes of TCR families in CD4^+^/CD8^+^ T cells in TB patient-47 (moderate group).

Our results showed that age-matched healthy controls had a normally diversified TCR repertoire while TB patients represent with restricted TCR repertoire. Patients with mild disease had the highest diversity of TCR repertoire while severely infected patients had the lowest, suggest TCR repertoire diversity had a significantly inversely correlates with disease severity, which was independent of other clinical parameters. In addition, TB patients showed preferred usage of certain TCR types and sequencing of the TCRs showed a high frequency usage of certain V and J gene segments and CDR3 motif. Data presented in this report promote a better understanding of the common characteristics of T cells among TB patients and provides new insight into the TCR repertoire associated with clinic presentation in TB patients.

## Materials and Methods

### Study Population

86 TB patients recruited from the Nanfang Hospital were assessed for eligibility (26 female, 60 male, mean age 43.77±14.36 years, range 18–74 years). No patient had a history of chronic lung disease or other diseases such as cancer, heart, kidney failure or autoimmune diseases, nor was they diagnosed with other bacterial or viral (include HIV) infections, and none of patients accepted chemotherapy or any other biological treatment in the past five years. TB diagnosis was made based on clinical signs and symptoms as well as chest X-ray (CXR) and acid-fast smear examination. Informed written consent was obtained from each patient before the start of the study, and the study was approved by the Ethics Committee at Southern Medical University.

**Figure 4:**
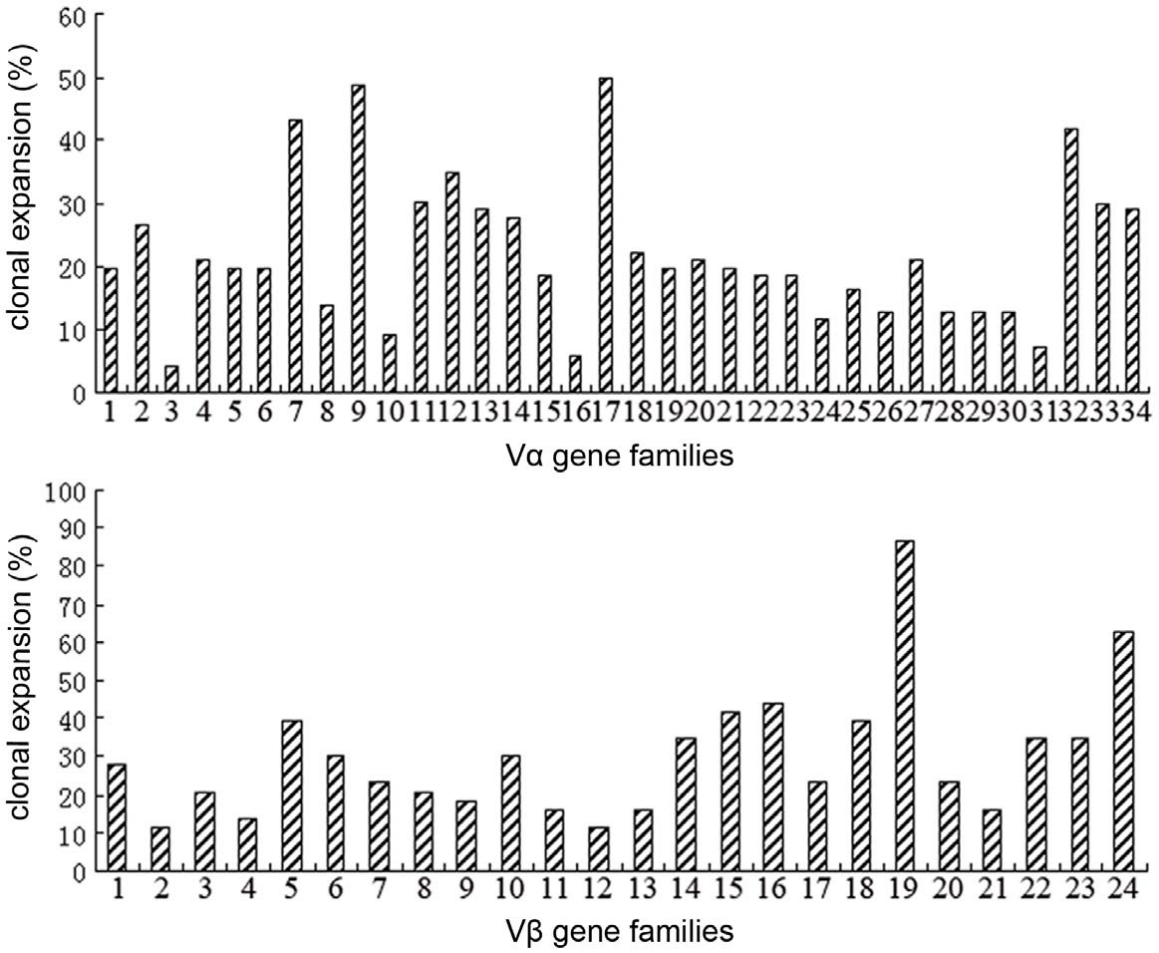
The preferred usage of TCR Vα and Vβ gene families. The percentage was calculated as the preferred usage frequency of corresponding gene family.

Two radiologists, blinded to patient’s clinical details, classification patients into mild, moderate and severe tuberculosis groups based on results of posteroanterior CXR (performed at the time of TB diagnosis). The classification criteria was that: the percentage of lung affected≤25%, effusion≤25% and no cavitation are determined as mild lesions; 25%<the percentage of lung affected≤50%, 25%<effusion≤50%, cavitation≤2 cm are determined as moderate lesions; the percentage of lung affected>50%, effusion>50%, cavitation>2 cm are determined as severe lesions.

Each patient’s clinical severity was also checked by two experienced pulmonologists based on integrated assessment of the sputum smear grade, radiographic result, clinical symptoms, and PPD skin test result. Then the 86 patients were divided into 3 groups: i) mild (n = 25, 16 males, 9 females, mean age of 44.0±14.04), ii) moderate (n = 28, 20 males, 8 females, mean age of 44.96±14.72) and iii) severe (n = 33, 24 males, 9 females, mean age of 42.58±14.63). T cells were obtained from blood samples before chemotherapy for the CDR3 spectratype analysis.

A group of 36 age-matched healthy blood donors was used to control for age and each patient was matched with a healthy group consisting of 3 healthy donors age-matched within 3 years. These donors had a BCG vaccination and had a positive PPD skin test (5∼15 mm). All of healthy blood donors had no abnormal chest radiographs and laboratory evidence of latent TB infection (determined by the antigen specific IFN-γ assay, T-SPOT.*TB*). All controls also had no clinical or laboratory evidence of connective tissue diseases or immunological disorders and were enrolled and consented as described above for the study population. Forty-three of the 86 patients and 11/36 healthy controls were smokers with a mean cumulative cigarette consumption of 5±4 pack-months and 3±2 packs/month, respectively.

**Table 2:** Conserved CDR3 sequences from α chain of T cells exhibited a single peak on CDR3 spectratype analysis.

Case	MHC Class I Haplotype	MHC Class II Haplotype	T cell	Vα	3′ Vα	N	5′ Proximal Jα
3	A*11∶01 A*26∶01	DRB1*08∶03 DRB1*15∶01	CD4^+^	Vα14	AVYFCAVR	GGGGGNKLI	FGTGTRLQVFP (**Jα34**)
7	A*11∶01 A*31∶01	DRB1*03∶01 DRB1*13∶12	CD8^+^	Vα31	ATYFCLLG	GGTGGNKLI	FGTGTRLQVFP (**Jα34**)
8	A*02∶07 A*24∶02	DRB1*04∶05 DRB1*15∶01	CD4^+^	Vα7	ATYLCAVK	GGAGGNKLI	FGKGTKLTVNP (Jα57)
11	A*01∶01 A*02∶07	DRB1*12∶01 DRB1*15∶01	CD8^+^	Vα15	AVYYCAVR	GGGGGNKLI	FGNGTKLTVNP (Jα57)
16	A*11∶01 A*11∶53	DRB1*08∶03 DRB1*12∶02	CD4^+^	Vα11	ATYFCAVR	GGGGGNKLI	FGPGTRLQVTL (Jα44)
17	A*11∶01 A*11∶01	DRB1*04∶06 DRB1*08∶03	CD8^+^	Vα3	AVYYCATD	GGGGNKLI	FGPGTRLLVRP (Jα12)
24	A*02∶07 A*33∶03	DRB1*13∶02 DRB1*14∶54	CD4^+^	Vα22	ATYLCLVGD	RGGGNNEQY	FGKGTSLTVNP (Jα54)
26	A*02∶01 A*02∶07	DRB1*04∶04 DRB1*12∶02	CD8^+^	Vα11	ASYLCAVE	GGGNDEQF	FGAGTRLTVKP (Jα43)
29	A*02∶07 A*11∶01	DRB1*09∶01 DRB1*09∶01	CD4^+^	Vα3	AIYLCAAS	RGGGNNEQF	FGKGTRLHILP (Jα30)
35	A*11∶01 A*30∶01	DRB1*04∶06 DRB1*12∶02	CD8^+^	Vα10	AAYYCAGE	GGTNEQF	FGTGTRLQVFP (**Jα34**)
37	A*02∶01 A*24∶02	DRB1*04∶10 DRB1*11∶01	CD4^+^	Vα19	ATYLCAVW	GTSNEQF	FGPGTRLFVKA (Jα9)
41	A*02∶07 A*24∶02	DRB1*14∶54 DRB1*16∶02	CD8^+^	Vα21	ATYFCAAS	GGGGHNEQY	FGTGTRLQVFP (**Jα34**)
42	A*02∶03 A*11∶53	DRB1*12∶02 DRB1*15∶02	CD4^+^	Vα25	AIYLCAGS	APDTGSGAF	FGPGTRLHILP (Jα30)
45	A*02∶01 A*02∶03	DRB1*11∶01 DRB1*12∶02	CD8^+^	Vα19	ALYFCGTV	APDTGSGAF	FGTGTRLQVFP (**Jα34**)
47	A*11∶01 A*24∶02	DRB1*08∶03 DRB1*09∶01	CD4^+^	Vα20	ASYLCLVGD	APDTGSGAF	FGQGTRLTVHP (Jα53)
56	A*02∶01 A*11∶01	DRB1*08∶03 DRB1*16∶02	CD8^+^	Vα19	ATYLCAVR	APDTGSGAF	FGQGTRLTIIP (Jα4)
62	A*11∶01 A*11∶01	DRB1*09∶01 DRB1*13∶12	CD8^+^	Vα7	ATYFCAVR	APDTGSGAF	FGPGTRVLVKP (Jα17)
71	A*02∶07 A*02∶07	DRB1*09∶01 DRB1*09∶01	CD8^+^	Vα19	AAYYCAVR	APDTGSGAF	FGPGTRLLVRP (Jα12)
73	A*02∶07 A*33∶03	DRB1*07∶01 DRB1*16∶02	CD4^+^	Vα31	AIYLCALD	APDTGSGAF	FGTGTRLQVFP (**Jα34**)
80	A*11∶01 A*11∶01	DRB1*09∶01 DRB1*15∶01	CD8^+^	Vα7	ASYLCAVR	APDTGSGAF	FGPGTRLSVKP (Jα14)

**Table 3:** Conserved CDR3 sequences from β chain of T cells exhibited a single peak on CDR3 spectratype analysis.

Case	MHC Class I Haplotype	MHC Class IIHaplotype	T cell	Vβ	3′ Vβ	N-D-N	5′ Proximal Jβ
5	A*01∶01 A*24∶02	DRB1*04∶03 DRB1*07∶01	CD4^+^	Vβ19	AVYLCASS	GGGSNKLI	YFGPGTRLTVT(J2S7)
9	A*01∶01 A*11∶01	DRB1*04∶03 DRB1*10∶01	CD8^+^	Vβ2	AVYLCASS	QGGSNKLI	QFFGPGTRLTVL(**J2S1**)
16	A*11∶01 A*11∶53	DRB1*08∶03 DRB1*12∶02	CD4^+^	Vβ20	AVYLCASS	GGGSNKLI	EQYFGPGTRLTVT(J2S7)
18	A*02∶01 A*11∶01	DRB1*04∶04 DRB1*14∶54	CD8^+^	Vβ5	AVYLCASS	GGGNKLI	EQFFGPGTRLTVL(**J2S1**)
31	A*11∶01 A*24∶02	DRB1*04∶03 DRB1*16∶02	CD4^+^	Vβ23	AVYLCASS	GGGANKLI	TQYFGPGTRLLVL(J2S5)
36	A*02∶07 A*11∶01	DRB1*09∶01 DRB1*14∶54	CD8^+^	Vβ8	AVYLCASS	GGGAKLI	FGPGTRLTVLX(**J2S1**)
40	A*02∶01 A*02∶07	DRB1*10∶01 DRB1*12∶02	CD4^+^	Vβ18	AVYLCASS	GGDNKLI	QYFGPGTRLTVL(J2S3)
45	A*02∶01 A*02∶03	DRB1*11∶01 DRB1*12∶02	CD8^+^	Vβ20	AVYLCASS	ASGTNKHI	FGNGTRLTVT(J1S6)
49	A*03∶01 A*33∶03	DRB1*03∶01 DRB1*13∶01	CD4^+^	Vβ24	AVFLCASR	DDGTNKLI	YNEQFFGPGTRLTVL(**J2S1**)
51	A*02∶07 A*24∶02	DRB1*09∶01 DRB1*12∶02	CD8^+^	Vβ3	AVYFCASS	EGCTTNKLI	FGDGTRLSIL(J1S5)
55	A*02∶07 A*24∶02	DRB1*14∶05 DRB1*15∶01	CD4^+^	Vβ11	AVYLCASS	EASTNKFI	NEQFFGPGTRLTVL(**J2S1**)
67	A*02∶07 A*11∶01	DRB1*04∶04 DRB1*12∶01	CD8^+^	Vβ6	AVYFCASS	GAASTNKLT	EQFFGPGTRLTVL(**J2S1**)
71	A*02∶07 A*02∶07	DRB1*09∶01 DRB1*09∶01	CD4^+^	Vβ8	AVYFCASS	GGSADKLI	FGNGTRLTVT(J1S6)
79	A*11∶01 A*24∶02	DRB1*08∶09 DRB1*12∶02	CD8^+^	Vβ10	AVYLCASS	ETSADKLI	FFGQFFGQGTRLTVV(J1S1)
81	A*11∶01 A*11∶01	DRB1*11∶01 DRB1*12∶02	CD4^+^	Vβ3	AVYLCASS	ETSDNKLI	NNEQFFGPGTRLTVL(**J2S1**)
84	A*02∶01 A*02∶06	DRB1*04∶05 DRB1*12∶02	CD8^+^	Vβ2	AVYLCASS	EITSGNKFI	QYFGPGTRLTVT(J2S7)

### Analysis of CDR3 Spectratype by GeneScan

Peripheral blood mononuclear cells (PBMCs) isolation, CD4^+^ and CD8^+^ T cell separation, CDR3 spectratype analysis, and use of the GeneScan CDR3 spectratype complexity scoring system was performed as previously described [21], [22]. To assess the reliability of the CDR3 spectratype complexity score system, samples drawn from the same individual at least two times were analyzed. The results demonstrated that the score system is quite reliable. We used relative complexity score (patient’s CDR3 spectratype complexity score / mean value of age-matched healthy donor group’s complexity score) to control for age variability when compare the TCR repertoire diversity among 3 patient groups.

### Sequencing the CDR3 Corresponding to TCR Vα and Vβ Families

The TCR Vα or TCR Vβ families showing CDR3 spectratype with single peaks following GeneScan analysis was selected to sequence since single peaks of defined CDR3 lengths often indicate monoclonal T cell expansions according to the specific characteristics of the hypervariable NDN-regions [23]. PCR products were amplified using the same TCR Vα or TCR Vβ family sense primers and TCR Cα or Cβ anti-sense primers (not FAM-labeled) using PCR conditions similar to those used for the first amplification. The PCR products will purified by gel electrophoresis and inserted into a pGEM-T vector (Promega Co., Madison, USA) and then sequence the T vector to obtain the nucleotide sequences of CDR3 to avoid measuring impassability in case of the single peak is reflected by two or more different NDN regions which have identical lengths. Nucleotide sequences of the amplified products were determined using an ABI 377 DNA sequencer (Applied Biosystems, Foster City, CA).

**Table 4:** The correlation of relative complexity scores and clinical parameters.

clinical parameters	*P*-values				*r*-values			
	Vα		Vβ		Vα		Vβ	
	CD4^+^	CD8^+^	CD4^+^	CD8^+^	CD4^+^	CD8^+^	CD4^+^	CD8^+^
disease severity	*0.000	*0.000	*0.000	*0.000	−0.823	−0.531	−0.455	−0.219
disease types	*0.014	0.825	0.441	*0.005	−0.264	−0.024	0.084	−0.297
gender	0.412	0.479	0.440	0.217	0.09	−0.077	0.084	−0.134
BCG vaccine history	0.584	0.973	0.357	0.188	−0.060	0.004	0.101	0.143
tuberculin skin test results	0.124	0.554	0.277	0.188	−0.167	0.065	0.119	0.143
previous TB or TB contact history	0.839	0.956	0.828	0.184	0.022	0.006	−0.024	0.145
smoking status	0.354	0.818	0.612	0.075	−0.101	−0.025	0.056	0.193

* *P*-values <0.05 considered has a statistically significant determined by spearman correlation test.

### Statistical Analyses

The paired t test was used to compare the means of the CDR3 spectratype complexity scores obtained from patients and healthy controls as well as CD4^+^ and CD8^+^ T cell subsets. One-way analysis of variance (One-way ANOVA) was used to test for relative complexity score differences among the 3 patient groups. K independent samples test was used to assess differences between patient age, gender, BCG vaccine history, tuberculin skin test results, previous TB or TB contact history, smoking status, and type of TB. Correlations between relative complexity scores and disease severity, as well as between relative scores and other patient characteristics were analyzed by spearman correlation test. All reported P-values were two-sided and *P*-values <0.05 were considered statistically significant. Statistical analyses were performed using the SPSS version 16.0 for windows statistical package (SPSS, Chicago, IL, USA).

## Results

### Demographic and Clinical Details

Demographic and clinical characteristics, as well as CDR3 spectratype complexity scores data for the TB patients are shown in Table 1.

### T Cell Clonality in TB Patients

We compared the CDR3 spectratypes of the 86 TB patients before treatment to the CDR3 spectraypes of the 36 age-matched healthy (uninfected) controls. Results demonstrated that most healthy controls displayed a normally diversified TCR repertoire based on the CDR3 spectratypes of their TCR Vα and Vβ gene sequences which showed a Gaussian distribution involving about 8 peaks (Fig. 1). The 9 elderly, age-matched healthy controls (age>60) presented with 4–6 oligoclonal subfamilies and 1–3 monoclonal subfamilies. In contrast, only rarely did TB patients present with a normal spectratype pattern, that is, the vast majority of TB patients presented with a restricted TCR repertoire by most of TCR Vα and Vβ gene families’ CDR3 spectratype showing fewer than 8 peaks or even just a single peak (Figure 1) Representative spectratype profiles of patients are shown in Figure 2 and Figure 3.

Analysis of T cell clonality among the 86 TB patients revealed that a particular Vα and Vβ TCR type prevailed, that is, Vα7 (43.02%), Vα9 (48.84%), Vα17 (50%), Vα32 (41.86%), Vβ19 (86.5%) and Vβ24 (62.79%) showed preferred usage more frequently than other families (Figure 4).

### Sequence Analysis of the TCR α and β Chain CDR3 Regions

PCR products from the TCR Vα and Vβ gene families showing CDR3 spectratype with single peaks following GeneScan analysis were selected for sequencing. This analysis identified a high frequency use of J gene segments Jα34, Jβ2-1, and of N nucleotide insertions in both α- and β-chains in these TB patients, that is, a highly conserved GGGGNKLI, GGGNEQYF, APDTGSGAF amino acid motif in the CDR3 of TCR α chains and GGGNKLI, TNKLI, SADKLI motif in the β chains. We also examined the MHC class I and class II haplotypes of the TB patients with conserved CDR3 motifs. This analysis did not identify a correlation between HLA haplotypes and conserved CDR3 motif usage (Table 2 and Table 3).

### TB Patient Spectratype Complexity Scores

The results of 86 TB patients obtained from the examination before chemotherapy was included for comparison of the TCR repertoire diversity between TB patients and age-matched healthy controls. CDR3 scoring system was used to obtain a uniform standard to quantify TCR repertoire diversity. The CDR3 spectratype complexity score for each patient was lower than that of the corresponding age-matched healthy controls. The mean scores for both CD4^+^ (230.80±17.84 *vs.* 262.65±0.97 for Vα, *P* = 0.000; 167.62±6.19 *vs.* 171.63±4.52 for Vβ, *P* = 0.000) and CD8^+^ T cells (206.66±23.17 *vs.* 249.93±4.01, for Vα, *P* = 0.000; 150.42±12.44 *vs.* 167.99±6.51 for Vβ, *P* = 0.000) in TB patients were significantly lower than those of age-matched healthy controls. The complexity scores of CD4^+^ T cells were significantly higher than those of CD8^+^ T cells both in healthy controls and in TB patients (*P* = 0.000).

### Relationship between the TCR Repertoire Diversity and Clinical Severity

It was previously reported that older, healthy individuals had a restricted T cell repertoire [24], [25]. To control for the influence of age, each patient was matched with a healthy group consisting of 3 healthy donors age-matched within 3 years. The ratio of the scores of the patient to that of the mean scores of the age-matched healthy group was used as a parameter to compare the TCR repertoire diversity among patient groups with different disease severities.

Patients in the mild group were found to have the highest relative complexity scores for both CD4^+^ T cell subsets. In contrast, the severe group had the lowest relative scores for both CD4^+^ and CD8^+^ T cells (Table 1). The relative complexity scores (TCR repertoire diversity) showed a significant negative correlation with disease severity, and have a certain degree of correlation with disease types. Importantly, comparisons between complexity scores for patients in the 3 groups revealed that other clinical parameters, including gender, BCG vaccine history, tuberculin skin test results, previous TB or TB contact history and smoking status could not account for the observed differences in TCR repertoire diversity (Table 4).

## Discussion

T lymphocytes have been shown to play critical roles in mediating anti-TB immune responses. Specifically, protective immunity against Mtb involves both CD4^+^ and CD8^+^ T cells. A majority of circulating mature T cells recognizes TB antigens via α/β TCRs. The α/β TCR repertoire diversity can be affected by CD4^+^ and CD8^+^ T cell clonal expansion following a TB infection that affects a patient’s immune response to this pathogen and in turn, disease severity. We evaluated the diversity of CD4^+^ and CD8^+^ T lymphocytes in TB patients in an attempt to discover the relationship between T cell repertoire diversity and disease presentation.

We demonstrated that CD4^+^ and CD8^+^ T lymphocyte expansion followed Mtb infection and was accompanied by the preferential expression of certain TCR Vα and Vβ gene families, supporting the previous work that demonstrated clonal expansion of Vβ 2^+^ CD8^+^ effector T cells in the peripheral blood of pediatric tuberculosis patients [20] and selection expansion of HLA-DR17, DQ2-restricted Vα 2.3^+^ CD4^+^ T cells following stimulation with live Mtb or soluble Mtb extracts [26]. Although the Vα and Vβ family expansion profiles differed between patients in our study, particular types of Vα and Vβ TCR gene families showed preferred usage more frequently than other gene families in TB patients, suggesting a common antigen recognize specificity in the TB patients.

To better understand the share nature of the T cells in disease presentation associated with Mtb infections, we sequenced the CDR3 of the clonally expanded T cells and found a highly conserved GGGGNKLI, GGGNEQYF, APDTGSGAF amino acid motif in the CDR3 of TCR α chains and GGGNKLI, TNKLI, SADKLI motif in the β chains as well as an increased frequency of Jα34 and Jβ2s7 in the proximal region of the TCR CDR3 which may be linked with certain common Mtb antigen identify attributes and will facilitate us to development of allogeneic adoptive T cell immunotherapy.

The variability of the expanded V families between individuals stimulation by Mtb 16-kDa antigen was previously described in the context of HLA polymorphisms [27]. However, no evidence demonstrated that similarly expanded Vα or Vβ family clones, or the conserved CDR3 sequence, occurred in patients with similar HLA backgrounds and cross-reactive TCR responses had been reported presented by different MHC class II molecules [28]. To determine whether the conserved CDR3 sequence was associated with a common HLA phenotype, we determined the HLA types of patients with conserved CDR3 sequences. The results indicated that conserved CDR3 sequences were independent of the HLA phenotype, suggesting that common TCR structural features exists in TB-associated T cells even in individual with different HLA phenotype.

Although TCR repertoire drift has been described to occur in peripheral blood samples collected from a small TB patient sample, previous studies ignore the influence of disease presentation to TCR repertoire diversity. Because the formation of TCR repertoire diversity was influenced by many factors, the extent of the likely contribution of TCRs specific to non-TB antigens is not known. So it is critical to reduce of the impact of TCR repertoire diversity by other factors as far as possible. We select TB patients with no history of chronic lung disease or other system diseases and without other bacterial or viral (include HIV) infections to decrease and testing patients prior to chemotherapy for chemotherapy can substantially change the TCR repertoire.

Mtb infection with high bacterial loads may changed the T cell immune response by i) anergy of skin DTH and T cell proliferation, ii) shifts the immune response from Th1 to Th2, and consequently, iii) enhanced B cell differentiation and antibody responses, which may associated with a more restricted TCR repertoire. Theoretically speaking, the severe patients load more Mtb bacterium than patients with mild or moderate TB [29]. Consistent with these, results described in this study demonstrated that TCR repertoire diversity correlated negatively with disease severity, that is, patients with mild disease had the highest relative complexity while severely infected patients had the lowest complexity. The TCR repertoire diversity showed a certain degree of correlation with disease types maybe because there is a correlation between certain TB types and disease severity. For example, tuberculosis pleurisy usually represents a mild type of TB which is generally resolves without treatment, and hematogenous disseminated pulmonary tuberculosis is a severe type of TB which always acutely ill with high fever and is in danger of dying [30].

Due to limitations in the collection of blood samples of more patients at different stages of disease, our analysis was restricted to patients in pre-treatment. A study including a cohort with different disease stages would allow for the confirmation of the data presented in this report. However, it is important to note that this study provides the first analysis describing the correlation between disease severity and TCR repertoire diversity *in vivo*, thereby providing useful information that allows for a better understanding of anti-Mtb immune responses. In addition, the public characteristics of T cells among TB patients may be association with certain common (conserve) TB antigen. Future analysis the antigen/epitope specificity of TCRs contain conserved CDR3 motif will be our next step of work.

## Supporting Information

Table S1 **Patient Groupings and Medical Diagnoses (n = 86).**
(DOC)

Table S2 **Healthy control characteristics and CDR3 score (n = 36).**
(DOC)
