# Supplementary material for: Limited T Cell Receptor Repertoire Diversity in Tuberculosis Patients Correlates with Clinical Severity
Source: PLoS One. 2012 Oct 26;7(10):e48117. doi: 10.1371/journal.pone.0048117 (PMC3482216; doi:10.1371/journal.pone.0048117)
Supplement: Table S1 — Patient Groupings and Medical Diagnoses (n = 86). (DOC) [file pone.0048117.s001.doc]

**Table S1. Patient Groupings and Medical Diagnoses (n=86).**

| Case | Sex | Age | Chest X-ray | Symptom | Pathography | Smoking status | Diagnosis | Group | patient’s CDR3 score | | | | age-match healthy group’s CDR3 score | | | | | Ratio of patient’s score /healthy’s score | | | | | |
| --- | --- | --- | --- | --- | --- | --- | --- | --- | --- | --- | --- | --- | --- | --- | --- | --- | --- | --- | --- | --- | --- | --- | --- |
|  |  |  |  |  |  |  |  |  | CD4 | | CD8 | | CD4 | | CD8 | | | CD4 | | | | CD8 | |
|  |  |  |  |  |  |  |  |  | Vα | Vβ | Vα | Vβ | Vα | Vβ | Vα | | Vβ | Vα | | Vβ | | Vα | Vβ |
| 1 | Male | 21 | No obvious abnormity | cough, expectoration | close contact with tuberculosis patients | light smoker | pulmonary tuberculosis | mild | 261 | 177 | 231 | 173 | 264.3 | 180 | | 259 | 179.3 | 0.99 | 0.98 | | 0.89 | | 0.96 |
| 2 | Male | 24 | Left pleural effusion | cough | no record | non smoker | tuberculous pleurisy | mild | 261 | 173 | 237 | 170 | 263 | 178.7 | | 256.7 | 177.3 | 0.99 | 0.97 | | 0.92 | | 0.96 |
| 3 | Female | 26 | Left pleural effusion | chest pain | no record | non smoker | tuberculous pleurisy | mild | 248 | 170 | 220 | 128 | 263 | 178.7 | | 256.7 | 177.3 | 0.94 | 0.95 | | 0.86 | | 0.72 |
| 4 | Female | 28 | Pleural effusion in right side | cough | no record | non smoker | tuberculous pleurisy | mild | 245 | 160 | 238 | 135 | 263.7 | 176.3 | | 252 | 172.3 | 0.93 | 0.91 | | 0.94 | | 0.78 |
| 5 | Female | 30 | Tuberculoma formation in upper lobes of both lung | expectoration | no record | non smoker | pulmonary tuberculosis | mild | 242 | 173 | 223 | 163 | 263.7 | 176.3 | | 252 | 172.3 | 0.92 | 0.98 | | 0.88 | | 0.95 |
| 6 | Female | 31 | Left pleural effusion | cough | no record | non smoker | tuberculous pleurisy | mild | 253 | 178 | 189 | 147 | 263.7 | 176.3 | | 252 | 172.3 | 0.96 | 1.01 | | 0.75 | | 0.85 |
| 7 | Female | 33 | Encapsulated effusion on the right chest | asymptomatic | no record | non smoker | tuberculous pleurisy | mild | 259 | 170 | 232 | 145 | 263.7 | 176.3 | | 252 | 172.3 | 0.98 | 0.96 | | 0.92 | | 0.84 |
| 8 | Male | 35 | Infiltrating pulmonary tuberculosis in lower left lung | chest pain, cough | no record | trivial smoker | pulmonary tuberculosis | mild | 258 | 171 | 224 | 171 | 263.3 | 173.3 | | 249.7 | 172 | 0.98 | 0.99 | | 0.9 | | 0.99 |
| 9 | Female | 37 | Upper right lung infiltrates, part scleroma | cough | no record | non smoker | pulmonary tuberculosis | mild | 264 | 176 | 217 | 168 | 263.3 | 173.3 | | 249.7 | 172 | 1 | 1.02 | | 0.87 | | 0.98 |
| 10 | Male | 39 | Infiltrating pulmonary tuberculosis on both lungs, part of the area presented with a scleroma | asymptomatic | no record | trivial smoker | pulmonary tuberculosis | mild | 251 | 171 | 237 | 151 | 263 | 171 | | 249 | 169.3 | 0.95 | 1 | | 0.95 | | 0.89 |
| 11 | Male | 39 | Tuberculosis and cavitation in upper right lung | chest pain, cough, blood in phlegm | no record | ex-trivial smoker | chronic fibrocavitative pulmonary tuberculosis | mild | 253 | 172 | 217 | 154 | 263 | 171 | | 249 | 169.3 | 0.96 | 1.01 | | 0.87 | | 0.91 |
| 12 | Male | 41 | No obvious abnormity | cough | no record | non smoker | pulmonary tuberculosis | mild | 250 | 175 | 221 | 143 | 263 | 171 | | 249 | 169.3 | 0.95 | 1.02 | | 0.89 | | 0.84 |
| 13 | Male | 42 | Encapsulated effusion on the left chest | asymptomatic | no record | trivial smoker | tuberculous pleurisy | mild | 232 | 174 | 182 | 164 | 263 | 171 | | 249 | 169.3 | 0.88 | 1.02 | | 0.73 | | 0.97 |
| 14 | Male | 43 | Infiltrating pulmonary tuberculosis in upper right lung | chest pain, cough | no record | ex-trivial smoker | pulmonary tuberculosis | mild | 235 | 173 | 223 | 144 | 262.7 | 170 | | 248.3 | 167.7 | 0.89 | 1.02 | | 0.9 | | 0.86 |
| 15 | Female | 45 | Part of encapsulated pleural effusion on the left side | cough | no record | non smoker | tuberculous pleurisy | mild | 246 | 170 | 222 | 162 | 262.7 | 170 | | 248.3 | 167.7 | 0.94 | 1 | | 0.89 | | 0.97 |
| 16 | Male | 47 | Upper left lung infiltrates, part scleroma | asymptomatic | no record | trivial smoker | pulmonary tuberculosis | mild | 261 | 171 | 240 | 166 | 262.3 | 169 | | 248 | 166.7 | 1 | 1.01 | | 0.97 | | 1 |
| 17 | Female | 51 | Moderate amount of pleural effusion on the right chest | asymptomatic | no record | non smoker | tuberculous pleurisy | mild | 240 | 170 | 228 | 158 | 262.3 | 169 | | 248 | 166.7 | 0.91 | 1.01 | | 0.92 | | 0.95 |
| 18 | Male | 53 | Tuberculosis in left lung, upper lobe | cough | no record | non smoker | pulmonary tuberculosis | mild | 245 | 168 | 231 | 166 | 262 | 168.7 | | 247.7 | 163.7 | 0.94 | 1 | | 0.93 | | 1.01 |
| 19 | Female | 57 | Tuberculosis in right lung, upper lobe | asymptomatic | no record | non smoker | pulmonary tuberculosis | mild | 249 | 169 | 230 | 115 | 262 | 168 | | 247 | 161 | 0.95 | 1.01 | | 0.93 | | 0.71 |
| 20 | Male | 59 | Infiltrating pulmonary tuberculosis on upper portion of both lungs, part of the area presented with a scleroma | asymptomatic | no record | non smoker | pulmonary tuberculosis | mild | 248 | 146 | 229 | 160 | 262 | 168 | | 247 | 161 | 0.95 | 0.87 | | 0.93 | | 0.99 |
| 21 | Male | 60 | Encapsulated effusion on the right chest | expectoration | no record | trivial smoker | tuberculous pleurisy | mild | 251 | 171 | 236 | 147 | 261.7 | 166.7 | | 246.7 | 160.7 | 0.96 | 1.03 | | 0.96 | | 0.91 |
| 22 | Male | 62 | Infiltrating pulmonary tuberculosis on lower lobe of both lungs, | asymptomatic | no record | ex-trivial smoker | pulmonary tuberculosis | mild | 245 | 174 | 194 | 131 | 261.7 | 166.7 | | 246.7 | 160.7 | 0.94 | 1.04 | | 0.79 | | 0.82 |
| 23 | Male | 63 | Moderate amount of pleural effusion on the left chest | chest pain | no record | light smoker | tuberculous pleurisy | mild | 242 | 162 | 187 | 148 | 261 | 166 | | 245.7 | 159 | 0.93 | 0.98 | | 0.76 | | 0.93 |
| 24 | Male | 65 | Significant pleural effusion on the right chest | cough | no record | light smoker | tuberculous pleurisy | mild | 249 | 161 | 240 | 153 | 261 | 166 | | 245.7 | 159 | 0.95 | 0.97 | | 0.98 | | 0.96 |
| 25 | Male | 69 | Encapsulated effusion on the left chest | expectoration | no record | trivial smoker | tuberculous pleurisy | mild | 245 | 169 | 208 | 150 | 260.3 | 165.7 | | 244.7 | 154.7 | 0.94 | 1.02 | | 0.85 | | 0.97 |
| 26 | Male | 19 | Pleural effusion in right side, right pleural thickening | intermittent fever, cough, expectoration | no record | non smoker | tuberculous pleurisy | moderate | 241 | 167 | 229 | 146 | 264.3 | 180 | | 259 | 179.3 | 0.91 | 0.93 | | 0.88 | | 0.81 |
| 27 | Male | 21 | Effusion on the right chest | asymptomatic | no record | non smoker | tuberculous pleurisy | moderate | 241 | 177 | 224 | 159 | 264.3 | 180 | | 259 | 179.3 | 0.91 | 0.98 | | 0.86 | | 0.89 |
| 28 | Female | 24 | Tuberculoma formation in upper lobes of both lung | asymptomatic | no record | non smoker | pulmonary tuberculosis | moderate | 245 | 179 | 184 | 163 | 263 | 178.7 | | 256.7 | 177.3 | 0.93 | 1 | | 0.72 | | 0.92 |
| 29 | Female | 28 | Pleural effusion on the left lung, left lower lung presenting with atelectasis | bosom frowsty, chest pain, cough | no record | non smoker | tuberculous pleurisy | moderate | 231 | 165 | 202 | 168 | 263.7 | 176.3 | | 252 | 172.3 | 0.88 | 0.94 | | 0.8 | | 0.98 |
| 30 | Male | 30 | Pleural effusion in right side | asymptomatic | no record | ex-trivial smoker | pulmonary tuberculosis | moderate | 237 | 171 | 228 | 168 | 263.7 | 176.3 | | 252 | 172.3 | 0.9 | 0.97 | | 0.9 | | 0.98 |
| 31 | Male | 32 | Part of encapsulated pleural effusion on the right side | asymptomatic | no record | non smoker | tuberculous pleurisy | moderate | 247 | 168 | 231 | 140 | 263.7 | 176.3 | | 252 | 172.3 | 0.94 | 0.95 | | 0.92 | | 0.81 |
| 32 | Male | 32 | The upper right lung with tuberculosis infiltrates, part of the scleroma, tuberculoma formation | asymptomatic | no record | ex-trivial smoker | pulmonary tuberculosis | moderate | 244 | 173 | 233 | 169 | 263.7 | 176.3 | | 252 | 172.3 | 0.93 | 0.98 | | 0.92 | | 0.98 |
| 33 | Female | 33 | Moderate amount of pleural effusion on the left chest | asymptomatic | no record | non smoker | pulmonary tuberculosis | moderate | 235 | 173 | 223 | 144 | 263.7 | 176.3 | | 252 | 172.3 | 0.89 | 0.98 | | 0.88 | | 0.84 |
| 34 | Female | 34 | tuberculosis in right lung, upper lobe | chest pain, cough | no record | non smoker | pulmonary tuberculosis | moderate | 236 | 173 | 196 | 152 | 263.3 | 173.3 | | 249.7 | 172 | 0.9 | 1 | | 0.78 | | 0.88 |
| 35 | Male | 39 | Tuberculosis on left lung, pleural effusion on the right chest | asymptomatic | no record | trivial smoker | pulmonary tuberculosis | moderate | 222 | 172 | 187 | 166 | 263 | 171 | | 249 | 169.3 | 0.84 | 1.01 | | 0.75 | | 0.98 |
| 36 | Female | 40 | Infiltrating pulmonary tuberculosis on left upper lung, tuberculoma formation,  tuberculosis lesions in upper right lung | chest pain, cough | no record | ex-trivial smoker | pulmonary tuberculosis | moderate | 232 | 172 | 130 | 165 | 263 | 171 | | 249 | 169.3 | 0.88 | 1.01 | | 0.52 | | 0.97 |
| 37 | Male | 41 | Moderate amount of pleural effusion on the left chest | fever, chest pain | no record | non smoker | tuberculous pleurisy | moderate | 233 | 166 | 199 | 141 | 263 | 171 | | 249 | 169.3 | 0.89 | 0.97 | | 0.8 | | 0.83 |
| 38 | Female | 41 | Bronchiectasis and infection inside the middle section of right lung, upper and lower lobe of left lung, multiple spots scattered shadows in both lung | cough, expectoration | acid-fast bacillus（+） | non smoker | pulmonary tuberculosis, bronchiectasia | moderate | 231 | 173 | 192 | 145 | 263 | 171 | | 249 | 169.3 | 0.88 | 1.01 | | 0.77 | | 0.86 |
| 39 | Male | 43 | Upper right lung infiltrates, part scleroma | asymptomatic | no record | non smoker | pulmonary tuberculosis | moderate | 232 | 163 | 195 | 143 | 262.7 | 170 | | 248.3 | 167.7 | 0.88 | 0.96 | | 0.79 | | 0.85 |
| 40 | Male | 45 | No obvious abnormity | chest pain | pathologic biopsy revealed scrofulous | light smoker | infiltrates tuberculosis, lymphatic tuberculosis | moderate | 213 | 171 | 199 | 142 | 262.7 | 170 | | 248.3 | 167.7 | 0.81 | 1.01 | | 0.8 | | 0.85 |
| 41 | Female | 47 | Moderate amount of pleural effusion on the left chest | dyspnea, cough | hydrothorax | non smoker | Tuberculous pleurisy | moderate | 228 | 169 | 206 | 141 | 262.3 | 169 | | 248 | 166.7 | 0.87 | 1 | | 0.83 | | 0.85 |
| 42 | Male | 48 | Large pleural effusion in right chest | asymptomatic | no record | trivial smoker | pulmonary tuberculosis | moderate | 230 | 168 | 215 | 151 | 262.3 | 169 | | 248 | 166.7 | 0.88 | 0.99 | | 0.87 | | 0.91 |
| 43 | Male | 49 | Space-occupying lesions in upper right lung | cough, blood in phlegm | pathologic biopsy revealed upper right lung tuberculosis | light smoker | pulmonary tuberculosis, lymphatic tuberculosis | moderate | 238 | 169 | 214 | 158 | 262.3 | 169 | | 248 | 166.7 | 0.91 | 1 | | 0.86 | | 0.95 |
| 44 | Male | 53 | Moderate amount of pleural effusion on the right chest | chest pain, cough | no record | ex-trivial smoker | tuberculous pleurisy | moderate | 222 | 171 | 197 | 162 | 262 | 168.7 | | 247.7 | 163.7 | 0.85 | 1.01 | | 0.8 | | 0.99 |
| 45 | Male | 58 | Infiltrating pulmonary tuberculosis on left upper lung | asymptomatic | no record | light smoker | pulmonary tuberculosis | moderate | 233 | 166 | 203 | 137 | 262 | 168 | | 247 | 161 | 0.89 | 0.99 | | 0.82 | | 0.85 |
| 46 | Female | 58 | infiltrating tuberculosis in left lung with cavitation | bosom frowsty, polypnea, | no record | non smoker | chronic fibrocavitative pulmonary tuberculosis | moderate | 237 | 159 | 228 | 153 | 262 | 168 | | 247 | 161 | 0.9 | 0.95 | | 0.92 | | 0.95 |
| 47 | Male | 59 | Lesions in upper and lower lobe of right lung, tuberculosis | dyspnea | no record | light smoker | tuberculous pleurisy | moderate | 241 | 153 | 238 | 160 | 262 | 168 | | 247 | 161 | 0.92 | 0.91 | | 0.96 | | 0.99 |
| 48 | Male | 60 | Space-occupying lesions in upper lobe of right lung | fever, cough, expectoration | no record | light smoker | pulmonary tuberculosis | moderate | 224 | 166 | 205 | 147 | 261.7 | 166.7 | | 246.7 | 160.7 | 0.86 | 1 | | 0.83 | | 0.91 |
| 49 | Male | 60 | Lesions in upper and lower lobe of left lung, tuberculosis | chest pain, cough | no record | non smoker | tuberculous pleurisy | moderate | 216 | 165 | 202 | 154 | 261.7 | 166.7 | | 246.7 | 160.7 | 0.83 | 0.99 | | 0.82 | | 0.96 |
| 50 | Male | 63 | Infiltrating pulmonary tuberculosis on upper left lungs | cough | no record | non smoker | pulmonary tuberculosis | moderate | 233 | 158 | 225 | 131 | 261 | 166 | | 245.7 | 159 | 0.89 | 0.95 | | 0.92 | | 0.82 |
| 51 | Male | 64 | Significant pleural effusion on the left chest | polypnea, cough | hydrothorax,  pathological examination revealed large fibrinousexudate | non smoker | tuberculous pleurisy | moderate | 214 | 167 | 201 | 151 | 261 | 166 | | 245.7 | 159 | 0.82 | 1.01 | | 0.82 | | 0.95 |
| 52 | Male | 65 | Encapsulated effusion on the right chest | polypnea, nachtsschweiss | hydrothorax | trivial smoker | tuberculous pleurisy | moderate | 229 | 170 | 218 | 148 | 261 | 166 | | 245.7 | 159 | 0.88 | 1.02 | | 0.89 | | 0.93 |
| 53 | Male | 73 | Double pneumonia disease bronchiectasis in lower lobes of left lung | chest pain | acid-fast bacillus（+） | non smoker | pulmonary tuberculosis, bronchiectasia | moderate | 217 | 163 | 216 | 147 | 260.3 | 165.7 | | 244.7 | 154.7 | 0.83 | 0.98 | | 0.88 | | 0.95 |
| 54 | Male | 18 | Consolidation of lung tissue in right and left lower lobes, pleural effusion in right side | thoracic back pain, fever | acid-fast bacillus（+） | non smoker | tuberculous pleurisy | severe | 223 | 167 | 227 | 173 | 264.3 | 180 | | 259 | 179.3 | 0.84 | 0.93 | | 0.88 | | 0.96 |
| 55 | Female | 20 | Infiltrating pulmonary tuberculosis on left upper lung, cavitation in upper left lung | fever,  hemoptysis | no record | non smoker | chronic fibrocavitative pulmonary tuberculosis | severe | 222 | 176 | 187 | 162 | 264.3 | 180 | | 259 | 179.3 | 0.84 | 0.98 | | 0.72 | | 0.9 |
| 56 | Male | 21 | Tuberculosis on right lung, pleural effusion on the left chest | cough, expectoration, right chest pain | pathological examination revealed granulomatous inflammation of pleural cavity | trivial smoker | tuberculous pleurisy | severe | 223 | 173 | 235 | 144 | 264.3 | 180 | | 259 | 179.3 | 0.84 | 0.96 | | 0.91 | | 0.8 |
| 57 | Male | 22 | Double upper lobe tuberculosis, atelectasis in middle lobe of right lung, right encapsulated pleural effusion after drainage, bilateral pleural thickening and adhesion | fever, polypnea, | acid-fast bacillus（++） | non smoker | pulmonary tuberculosis | severe | 225 | 173 | 211 | 144 | 264.3 | 180 | | 259 | 179.3 | 0.85 | 0.96 | | 0.81 | | 0.8 |
| 58 | Male | 22 | Lesions in upper and lower lobe of left lung, tuberculosis, and cavitation | cough, expectoration,  fever,  hemoptysis | no record | ex-trivial smoker | pulmonary tuberculosis | severe | 232 | 163 | 195 | 143 | 264.3 | 180 | | 259 | 179.3 | 0.88 | 0.91 | | 0.75 | | 0.8 |
| 59 | Female | 23 | Infiltrating pulmonary tuberculosis on double upper lung | asymptomatic | no record | non smoker | infiltrates tuberculosis | severe | 223 | 171 | 192 | 140 | 263 | 178.7 | | 256.7 | 177.3 | 0.85 | 0.96 | | 0.75 | | 0.79 |
| 60 | Male | 24 | Moderate amount of pleural effusion on the left chest | fever, cough, dyspnea | needle biopsy of pleura | light smoker | tuberculous pleurisy | severe | 231 | 176 | 233 | 141 | 263 | 178.7 | | 256.7 | 177.3 | 0.88 | 0.98 | | 0.91 | | 0.8 |
| 61 | Male | 24 | Infiltrating tuberculosis in right lung with cavitation | cough, hemoptysis | no record | trivial smoker | chronic fibrocavitative pulmonary tuberculosis | severe | 213 | 172 | 182 | 160 | 263 | 178.7 | | 256.7 | 177.3 | 0.81 | 0.96 | | 0.71 | | 0.9 |
| 62 | Female | 31 | Diffuse nodules shadow in both lungs | cough, expectoratio,  chest pain | acid-fast bacillus（+） | non smoker | hematogenous disseminated pulmonary tuberculosis | severe | 217 | 168 | 212 | 151 | 263.7 | 176.3 | | 252 | 172.3 | 0.82 | 0.95 | | 0.84 | | 0.88 |
| 63 | Male | 36 | Moderate pleural effusion in left chest, left upper lobe tuberculosis and tuberculoma formation | asymptomatic | no record | light smoker | tuberculous pleurisy, pulmonary tuberculosis | severe | 221 | 153 | 178 | 130 | 263.3 | 173.3 | | 249.7 | 172 | 0.84 | 0.88 | | 0.71 | | 0.76 |
| 64 | Male | 37 | Infiltrate tuberculosis in double upper lung, part of scleroma and cavitation formation in lower of right lung | asymptomatic | no record | ex-trivial smoker | chronic fibrocavitative pulmonary tuberculosis | severe | 210 | 161 | 196 | 153 | 263.3 | 173.3 | | 249.7 | 172 | 0.8 | 0.93 | | 0.78 | | 0.89 |
| 65 | Female | 40 | Large effusion in left chest | fever, cough, polypnea, | no record | non smoker | tuberculous pleurisy | severe | 223 | 170 | 170 | 128 | 263 | 171 | | 249 | 169.3 | 0.85 | 0.99 | | 0.68 | | 0.76 |
| 66 | Female | 41 | Right lung atelectasis，bilateral small amount of pleural effusion | chest pain, cough | needle biopsy of pleura,  acid-fast bacillus（+++） | non smoker | tuberculous pleurisy | severe | 216 | 166 | 167 | 131 | 263 | 171 | | 249 | 169.3 | 0.82 | 0.97 | | 0.67 | | 0.77 |
| 67 | Male | 41 | Infiltrating pulmonary tuberculosis on both upper lungs | bosom frowsty, dyspnea | no record | non smoker | infiltrates tuberculosis | severe | 206 | 170 | 167 | 169 | 263 | 171 | | 249 | 169.3 | 0.78 | 0.99 | | 0.67 | | 1 |
| 68 | Male | 41 | Space-occupying lesions in middle lobe of right lung | cough, expectoration,  fever | acid-fast bacillus（++） | light smoker | pulmonary tuberculosis | severe | 228 | 168 | 217 | 157 | 263 | 171 | | 249 | 169.3 | 0.87 | 0.98 | | 0.87 | | 0.93 |
| 69 | Female | 42 | No obvious abnormity by chest X-ray, mediastinal lymph node enlargement by CT, lining tuberculosis by tracheal mirror | cough | tracheal mirror pathologic examination: tuberculosis | non smoker | infiltrates tuberculosis, endobronchial tuberculosis | severe | 223 | 166 | 221 | 156 | 263 | 171 | | 249 | 169.3 | 0.85 | 0.97 | | 0.89 | | 0.92 |
| 70 | Male | 42 | The upper right lung tuberculosis infiltrates | blood-stained sputum | no record | trivial smoker | pulmonary tuberculosis | severe | 208 | 170 | 164 | 156 | 263 | 171 | | 249 | 169.3 | 0.79 | 0.99 | | 0.66 | | 0.92 |
| 71 | Male | 46 | Inflammation of the inferior lobe of right lung with some tissue consolidation of lung tissue and inflammation in the left upper lobe, focal bronchiectasis in both lungs, pleural effusion in right side | fever | acid-fast bacillus（-）  pathological examination: chronic inflammation | Non smoker | infiltrates tuberculosis | severe | 234 | 161 | 184 | 140 | 262.7 | 170 | | 248.3 | 167.7 | 0.89 | 0.95 | | 0.74 | | 0.83 |
| 72 | Female | 47 | Infiltrating pulmonary tuberculosis on both lungs, cavitation formation | asymptomatic | no record | Non smoker | chronic fibrocavitative pulmonary tuberculosis | severe | 203 | 169 | 168 | 137 | 262.3 | 169 | | 248 | 166.7 | 0.77 | 1 | | 0.68 | | 0.82 |
| 73 | Male | 48 | Large effusion in right chest | fever, polypnea | no record | Trivial smoker | tuberculous pleurisy | severe | 231 | 165 | 187 | 141 | 262.3 | 169 | | 248 | 166.7 | 0.88 | 0.98 | | 0.75 | | 0.85 |
| 74 | Male | 49 | Moderate pleural effusion in right side | cough, expectoration | acid-fast bacillus（++） | Trivial smoker | tuberculous pleurisy | severe | 193 | 165 | 187 | 143 | 262.3 | 169 | | 248 | 166.7 | 0.74 | 0.98 | | 0.75 | | 0.86 |
| 75 | Male | 53 | Upper right tuberculosis, moderate pleural effusion in right chest | asymptomatic | no record | Non smoker | pulmonary tuberculosis | severe | 228 | 169 | 226 | 163 | 262 | 168.7 | | 247.7 | 163.7 | 0.87 | 1 | | 0.91 | | 1 |
| 76 | Male | 54 | Pleural effusion on the left chest and pleural hypertrophy | cough, hemoptysis, left chest pain | acid-fast bacillus（++） | Non smoker | pulmonary tuberculosis | severe | 175 | 154 | 184 | 120 | 262 | 168.7 | | 247.7 | 163.7 | 0.67 | 0.91 | | 0.74 | | 0.73 |
| 77 | Male | 54 | Double pneumonia, moderate pleural effusion in right side | paroxysmal right chest pain, cough | hydrothorax | Light smoker | pulmonary tuberculosis,  pleural pathology:  tuberculous pleurisy,  granulomatous inflammation | severe | 224 | 164 | 205 | 153 | 262 | 168.7 | | 247.7 | 163.7 | 0.85 | 0.97 | | 0.83 | | 0.93 |
| 78 | Male | 54 | Tuberculosis and cavitation formation in both lungs,  mediastinal and double hilar multiple lymph node | asymptomatic | no record | Trivial smoker | pulmonary tuberculosis | severe | 199 | 160 | 184 | 157 | 262 | 168.7 | | 247.7 | 163.7 | 0.76 | 0.95 | | 0.74 | | 0.96 |
| 79 | Male | 55 | Infiltrates in both lungs, moderate pleural effusion in right chest | bosom frowsty, polypnea, | no record | Trivial smoker | pulmonary tuberculosis | severe | 229 | 166 | 211 | 143 | 262 | 168.7 | | 247.7 | 163.7 | 0.87 | 0.98 | | 0.85 | | 0.87 |
| 80 | Male | 56 | Effusion on the right chest | fever, cough, expectoration | hydrothorax | Light smoker | tuberculous pleurisy | severe | 231 | 166 | 223 | 147 | 262 | 168.7 | | 247.7 | 163.7 | 0.88 | 0.98 | | 0.9 | | 0.9 |
| 81 | Male | 56 | Multiple density anomalies in the right lung, right pleural thickening | blood in phlegm | no record | Trivial smoker | pulmonary tuberculosis | severe | 219 | 161 | 216 | 147 | 262 | 168.7 | | 247.7 | 163.7 | 0.84 | 0.95 | | 0.87 | | 0.9 |
| 82 | Male | 57 | Effusion on the right chest | fever,  chest pain | hydrothorax | Light smoker | tuberculous pleurisy | severe | 179 | 158 | 156 | 148 | 262 | 168 | | 247 | 161 | 0.68 | 0.94 | | 0.63 | | 0.92 |
| 83 | Female | 58 | Large pleural effusion in left chest | asymptomatic | no record | Trivial smoker | pulmonary tuberculosis | severe | 228 | 163 | 199 | 160 | 262 | 168 | | 247 | 161 | 0.87 | 0.97 | | 0.81 | | 0.99 |
| 84 | Male | 59 | Infiltrating pulmonary tuberculosis on right upper lung | asymptomatic | hydrothorax | Trivial smoker | pulmonary tuberculosis | severe | 196 | 160 | 176 | 156 | 262 | 168 | | 247 | 161 | 0.75 | 0.95 | | 0.71 | | 0.97 |
| 85 | Female | 60 | Tuberculosis and cavitation formation in both lungs, moderate pleural effusion in right side | asymptomatic | no record | Non smoker | pulmonary tuberculosis | severe | 225 | 164 | 189 | 143 | 261.7 | 166.7 | | 246.7 | 160.7 | 0.86 | 0.98 | | 0.77 | | 0.89 |
| 86 | Male | 74 | Large pleural effusion in right side, nodules in upper right pulmonary | fever,  cough, chest pain | hydrothorax | Trivial smoker | tuberculous pleurisy | severe | 196 | 156 | 168 | 137 | 260.3 | 165.7 | | 244.7 | 154.7 | 0.75 | 0.94 | | 0.69 | | 0.89 |
