# Supplementary material for: Limited T Cell Receptor Repertoire Diversity in Tuberculosis Patients Correlates with Clinical Severity
Source: PLoS One. 2012 Oct 26;7(10):e48117. doi: 10.1371/journal.pone.0048117 (PMC3482216; doi:10.1371/journal.pone.0048117)
Supplement: Table S2 — Healthy control characteristics and CDR3 score (n = 36). (DOC) [file pone.0048117.s002.doc]

**Table S2．H**ealthy control characteristics and CDR3 score (n=36).

| Donor | Sex | Age | Smoking status | CDR3 score | | | | Age group | Average CDR3 score | | | |
| --- | --- | --- | --- | --- | --- | --- | --- | --- | --- | --- | --- | --- |
|  |  |  |  | CD4 | | CD8 | |  | CD4 | | CD8 | |
|  |  |  |  | Vα | Vβ | Vα | Vβ |  | Vα | Vβ | Vα | Vβ |
| 1 | Male | 74 | Trivial smoker | 260 | 166 | 245 | 150 | 69-74 | 260.3 | 165.7 | 244.7 | 154.7 |
| 2 | Female | 72 | Non smoker | 261 | 165 | 243 | 156 |  |  |  |  |  |
| 3 | Male | 69 | Non smoker | 260 | 166 | 246 | 158 |  |  |  |  |  |
| 4 | Male | 65 | Trivial smoker | 262 | 166 | 243 | 157 | 63-65 | 261 | 166 | 245.7 | 159 |
| 5 | Male | 64 | Non smoker | 261 | 165 | 248 | 159 |  |  |  |  |  |
| 6 | Female | 63 | Non smoker | 260 | 167 | 246 | 161 |  |  |  |  |  |
| 7 | Female | 61 | Non smoker | 263 | 166 | 245 | 160 | 60-62 | 261.7 | 166.7 | 246.3 | 160.7 |
| 8 | Male | 62 | Non smoker | 261 | 167 | 248 | 162 |  |  |  |  |  |
| 9 | Female | 60 | Non smoker | 261 | 167 | 246 | 160 |  |  |  |  |  |
| 10 | Male | 59 | Non smoker | 262 | 168 | 247 | 162 | 57-59 | 262 | 168 | 247 | 161 |
| 11 | Female | 57 | Non smoker | 263 | 167 | 246 | 161 |  |  |  |  |  |
| 12 | Male | 58 | Trivial smoker | 261 | 169 | 248 | 160 |  |  |  |  |  |
| 13 | Male | 56 | Trivial smoker | 263 | 169 | 248 | 165 | 53-56 | 262 | 168.7 | 247.7 | 163.7 |
| 14 | Male | 55 | Non smoker | 261 | 169 | 246 | 163 |  |  |  |  |  |
| 15 | Female | 53 | Non smoker | 262 | 168 | 249 | 163 |  |  |  |  |  |
| 16 | Male | 51 | Light smoker | 261 | 170 | 247 | 169 | 47-51 | 262.3 | 169 | 248 | 166.7 |
| 17 | Male | 49 | Trivial smoker | 262 | 169 | 248 | 167 |  |  |  |  |  |
| 18 | Female | 47 | Non smoker | 264 | 168 | 249 | 164 |  |  |  |  |  |
| 19 | Female | 46 | Non smoker | 262 | 168 | 246 | 166 | 43-46 | 262.7 | 170 | 248.3 | 167.7 |
| 20 | Female | 44 | Non smoker | 264 | 171 | 249 | 169 |  |  |  |  |  |
| 21 | Male | 43 | Trivial smoker | 262 | 171 | 250 | 168 |  |  |  |  |  |
| 22 | Female | 42 | Non smoker | 262 | 172 | 249 | 169 | 39-42 | 263 | 171 | 249 | 169.3 |
| 23 | Male | 41 | Light smoker | 264 | 170 | 247 | 171 |  |  |  |  |  |
| 24 | Female | 39 | Non smoker | 263 | 171 | 251 | 168 |  |  |  |  |  |
| 25 | Female | 37 | Non smoker | 263 | 173 | 246 | 171 | 34-37 | 263.3 | 173.3 | 249.7 | 172 |
| 26 | Female | 35 | Non smoker | 264 | 175 | 250 | 172 |  |  |  |  |  |
| 27 | Male | 34 | Light smoker | 263 | 172 | 253 | 173 |  |  |  |  |  |
| 28 | Female | 33 | Non smoker | 262 | 176 | 248 | 172 | 28-33 | 263.7 | 176.3 | 252 | 172.3 |
| 29 | Male | 30 | Trivial smoker | 264 | 177 | 257 | 171 |  |  |  |  |  |
| 30 | Male | 28 | Non smoker | 265 | 176 | 251 | 174 |  |  |  |  |  |
| 31 | Female | 26 | Non smoker | 264 | 177 | 253 | 176 | 23-26 | 263 | 178.7 | 256.7 | 177.3 |
| 32 | Male | 25 | Non smoker | 262 | 179 | 259 | 178 |  |  |  |  |  |
| 33 | Female | 23 | Non smoker | 263 | 180 | 258 | 178 |  |  |  |  |  |
| 34 | Male | 22 | Trivial smoker | 264 | 176 | 257 | 179 | 18-22 | 264.3 | 180 | 259 | 179.3 |
| 35 | Male | 20 | Non smoker | 266 | 181 | 259 | 179 |  |  |  |  |  |
| 36 | Female | 18 | Non smoker | 263 | 183 | 261 | 180 |  |  |  |  |  |
